# Effects of magnetically targeted iron oxide@polydopamine-labeled human umbilical cord mesenchymal stem cells in cerebral infarction in mice

**DOI:** 10.18632/aging.204540

**Published:** 2023-02-23

**Authors:** Jun Yan, Te Liu, Yang Li, Jun Zhang, Bo Shi, Fuqiang Zhang, Xuejia Hou, Xiaowen Zhang, Wanxing Cui, Jing Li, Hua Yao, Xiuying Li, Yufei Gao, Jinlan Jiang

**Affiliations:** 1 Department of Scientific Research Center, China-Japan Union Hospital of Jilin University, Changchun 130000, Jilin, China; 2 Central Laboratory, Dalian Municipal Women and Children’s Medical Center (Group), Xigang District, Dalian 116012, China; 3 Department of Anesthesiology, The First Hospital of Jilin University, Changchun 130000, Jilin, China; 4 Georgetown University Hospital, Washington, DC 20007, USA; 5 Department of Neurosurgery, China-Japan Union Hospital, Jilin University, Changchun 130031, Jilin, China

**Keywords:** human umbilical cord mesenchymal stem cell, cerebral infarction, iron oxide nanoparticle, inflammation

## Abstract

Mesenchymal stem cells are a potential therapeutic candidate for cerebral infarction due to their anti-inflammatory proprieties. However, ensuring the engraftment of sufficient cells into the affected brain area remains a challenge. Herein, magnetic targeting techniques were used for the transplantation of a large number of cells noninvasively. Mice subjected to pMCAO surgery were administered MSCs labeled or not with iron oxide@polydopamine nanoparticles by tail vein injection. Iron oxide@polydopamine particles were characterized by transmission electron microscopy, and labeled MSCs were characterized by flow cytometry and their differentiation potential was assessed *in vitro*. Following the systemic injection of iron oxide@polydopamine-labeled MSCs into pMCAO-induced mices, magnetic navigation increased the MSCs localization to the brain lesion site and reduced the lesion volume. Treatment with iron oxide@polydopamine-labeled MSCs also significantly inhibited M1 microglia polarization and increased M2 microglia cell infiltration. Furthermore, western blotting and immunohistochemical analysis demonstrated that microtubule-associated protein 2 and NeuN levels were upregulated the brain tissue of mice treated with iron oxide@polydopamine-labeled MSCs. Thus, iron oxide@polydopamine-labeled MSCs attenuated brain injury and protected neurons by preventing pro-inflammatory microglia activation. Overall, the proposed iron oxide@polydopamine-labeled MSCs approach may overcome the major drawback of the conventional MSCs therapy for the treatment of cerebral infarction.

## INTRODUCTION

In the last decade, cerebral infarction become the second leading cause of adult death and long-term disability worldwide, in particular in developing countries, which imposes a heavy financial burden on the affected individual as well as on the society [1–4]. Blood-vessel occlusion and subsequent neuronal damage is a main pathological event associated with cerebral infarction, of which neuroinflammation is a major consequence [2, 4]. Neuroinflammation, as a key mechanism behind secondary injury of cerebral infarction, is caused by dead cells and debris due to the infarction injury [5], and is characterized by microglia-induced peripheral leukocyte influx into the brain parenchyma and the release of proinflammatory cytokines. Indeed, microglia, which have long been considered one of the earliest and important participants in neuroinflammation of the central nervous system, is considered a critical factor in the inflammatory response after cerebral infarction [6, 7]. Altogether these events are detrimental to trigger and support a pro-inflammatory status in the brain microenvironment [8–10]. Therefore, investigations on the regulatory mechanisms of the inflammatory response during cerebral infarction has received more attention in recent years.

Mesenchymal stem cells (MSCs) are believed to hold therapeutic potential to ameliorate the effects of cerebral infarction owing to their multifaceted functions, such as secretion of numerous trophic factors that can modulate inflammation and angiogenesis [11], apoptosis [12, 13], and the immune response [12, 14–19]. It is generally believed that the ability of MSCs to target brain lesions and the number of functional MSCs transplanted can determine their therapeutic effectiveness [20]. The intracerebral implantation of cells into the infarcted brain is invasive and may cause additional damage to the healthy tissues. Intravenous administration is simpler and less invasive in comparison with direct implantation; however, only a small proportion of MSCs transplanted intravenously can effectively reach the brain lesion site *in vivo*, which hampers the clinical use of MSCs for treatment of cerebral infarction. Several strategies have been applied to enhance the migration and maintain the function of these cells at targeted sites. For example, genetic modifications have been used to overexpress receptors that recognize chemoattractants and promote cell migration, but only few of the injected MSCs are delivered to its targets [21]. Nonetheless, the migration efficiency of MSCs *in vivo* remains is unsatisfactory. Therefore, enhancing the homing strategy of MSCs to the ischemic brain may help enhance the treatment outcome. Magnetic iron oxide nanoparticles (MIONs) are a conventional magnetic resonance imaging contrast agent that holds application value for MSCs labeling. MIONs are approved for clinical use due to their pronounced biocompatibility, and have garnered increased attention because of their unique response features to external magnetic fields. Polydopamine (PDA) is highly biocompatible and biodegradable, and therefore, it is widely used to coat nanoparticles for numerous biomedical applications. Hence, PDA-capped Fe_3_O_4_ (MIONs@PDA) and their composites are among the safest nanomaterials used for clinical diagnosis and therapy. To obtain MSCs with a suitable targeting ability, magnetic MSCs were prepared using MIONs@PDA-coated MSCs (MIONs@PDA-MSCs). These nanoparticles contain Fe_3_O_4_ that mediates their magnetic navigation to the target brain infarction lesion with the assistance of an external magnetic field (MF).

The present study describes an animal model of permanent middle cerebral artery occlusion (pMCAO) that was established to explore the effects of MIONs@PDA-MSCs(MF) on cerebral infarction and assess the impact of MSCs in microglia activation and inflammation.

## MATERIALS AND METHODS

### Preparation of MIONs and transmission electron microscopy (TEM) analysis

MIONs were synthesized by the thermal decomposition method as previously described [22]. Briefly, the Fe_3_O_4_ nanoparticles were injected into sodium dodecyl sulfate (SDS, 99%), which was heated to obtain SDS-capped Fe_3_O_4_ super particles. Next, the separation of oleic acid stable nanoparticles was achieved using a magnet. The capped Fe_3_O_4_ nanoparticles were dispersed in Tris buffer (10 nM, pH = 8.5), which contained 6 mg/mL PDA aqueous solution and stirred for 3 h. The obtained MIONs@PDA were detected using an H-800 transmission electron microscope (Hitachi Ltd., Tokyo, Japan) with a charge coupled device camera.

### Culture, expansion, identification, and MIONs-labeling of human umbilical cord MSCs (HUMSCs)

Briefly, HUMSCs were obtained after normal deliveries following 38–40 week gestations. HUMSCs were cultured in Dulbecco’s modified Eagle medium (Gibco, Waltham, MA, USA) containing 10% fetal bovine serum (Gibco) and negative for mycoplasma contamination. All experiments were conducted using HUMSCs at passages 5–8. After reaching 80% confluency, various concentrations of MIONs@PDA (0, 10, 25, 50, 100, and 200 μg/mL) were added to the medium for 12 h. The Cell Counting Kit-8 (Sigma-Aldrich, St. Louis, MO, USA) assay was used to detect the cytotoxicity of MIONs@PDA. HUMSCs were stained with the Prussian blue iron staining kit (Solarbio, Beijing, China), according to the manufacturer’s instructions. The phenotype of HUMSCs was confirmed by the expression of surface markers (positive for CD44-fluorescein isothiocyanate (FITC) and CD105-phycoerythrin (PE), and negative for CD45-FITC) (all from BD Biosciences, San Jose, CA, USA) using a FACSC anto II flow cytometer (FC500; Beckman Coulter Brea, CA, USA), and the data were analyzed with the CXP software (Beckman Coulter). Differentiation capacity of the HUMSCs was assessed by inducing osteocyte and adipocyte differentiation using the StemPro Osteogenesis/Adipogenesis Differentiation Kit (Invitrogen, Waltham, MA, USA) and evaluated by Alizarin Red S, Oil red O, and Alcian blue staining, respectively.

### Mouse treatment and transplanted HUMSCs

Male C57/BL6 mice were purchased from the Beijing Weitong Lihua Experimental Animal Technology Co. (Beijing, China) and were housed in the animal facilities of the animal center of the College of Basic Medical Sciences, Jilin University, China.

The mice were anesthetized with 1.5% isoflurane (RWD Life Science, Shenzhen, China). Through a midline skin incision, the right common carotid artery (CCA), external carotid artery, and internal carotid artery (ICA) were isolated and ligated. Monofilament nylon suture was inserted from the right CCA to the ICA through a small incision in the CCA, and then advanced to the Circle of Willis to occlude the origin of the right middle cerebral artery. Subsequently, a silk suture was then tightened around the right common carotid artery stumps and nylon filament and then sutured the skin incision. Sham-operated mice underwent the same procedures except for the pMCAO. Behavioral evaluations of the mice were performed 24 h after surgery, using the Bederson 4-point rating scale scored as: 0, no deficit; 1, failing to stretch right forepaw during tail suspension test; 2, decreasing ability of forelimb resistance to contralateral thrust; and 3, circling to the right after holding the tail.The standard PMCAO model was defined as a Bederson scale score >1 point, and animals that did not meet this criterion were excluded from the study [23]. The rats were randomly divided into five groups (n = 5 in each group): non-treated sham, pMCAO surgery, and pMCAO treated with HUMSCs, MIONs@PDA-MSCs, or MIONs@PDA-MSCs(MF). A total of 5 × 10^5^ HUMSCs were injected through the tail vein 24 h after the surgery. The non-treated groups were given equal volume of phosphate-buffered saline (PBS).

### Calculation of infarct volume

Frozen sections were made every 2 mm along the sagittal axis of the brain. Then, TTC (2,3,5-triphenyl-2H-tetrazoliuM chloride) staining of tissue sections was carried out in a conventional manner. The percent infarct (mm^2^) was measured as follows: %_infarct_ = [(V_C_ − V_L_)/V_C_] × 100, where V_C_ and V_L_ represent the volume of the control hemisphere and the noninfarcted tissue in the lesioned hemisphere, respectively.

### Near-infrared fluorescence (NIRF) imaging

CM-Dil-labeled HUMSC were used for NIRF imaging. For the targeting study, 12 h and 5 d after intravenous HUMSCs administration, the mice were anesthetized and euthanized. An IVIS Spectrum imaging system (PerkinElmer, Waltham, MA, USA) was employed to capture the NIRF images, and the CM-Dil-related fluorescent signals were discriminated using the Living Image software (PerkinElmer).

### Histopathological, immunohistochemical (IHC), and immunofluorescence evaluation of brain tissues

The mice were decapitated and their organs (heart, liver, spleen, lung, kidney, and brain) were fixed in 4% paraformaldehyde overnight at 4° C and embedded in paraffin. The organs were sectioned into 5 μm thick pieces and partial dewaxing was immediately performed with xylene (5 mm tissue) followed by washing using a graded ethanol series (100%, 95%, 80%, and 75% diluted in distilled water). For histopathological examination, the samples were stained with hematoxylin (2 g/L) for 5 min and eosin (1%) for 2 min before washing with distilled water. For IHC analysis, the paraffin sections were blocked for 1 h and then incubated with antibodies against microtubule-associated protein 2 (MAP2) and NeuN (both at 1:200; ProteinTech, Chicago, IL, USA) at 4° C, overnight. After washing, the sections were incubated with a biotin-labeled secondary antibody and streptavidin-peroxidase for 30 min. Color development was achieved upon incubation with diaminobenzidine (MaiXin, Fuzhou, China), after which hematoxylin staining, dehydration, and neutral resin mounting were performed. For immunofluorescence, the sections were incubated with a fluorescent-dye-conjugated secondary antibody. Next, dehydration was carried out with absolute ethanol, and the tissue was sealed with a neutral resin. Images were collected at ×200 amplification with a microscope (Olympus Corporation, Tokyo, Japan).

### Quantitative real-time polymerase chain reaction (qRT-PCR) and Western blotting

Total RNA from the tissues corresponding to the lesion region was extracted using the Trizol reagent (Life Technologies, Waltham, MA, USA) and cDNA synthesis was performed using Reverse Transcriptase II (Invitrogen) according to the manufacturer’s instructions. QRT-PCR reactions were carried out in an ABI 7500 system in 10 μL reactions, with 1 μL cDNA samples and SYBR Premix ExTaq (TaKaRa, Kusatsu, Japan). Relative mRNA expression was calculated and analyzed using the comparative 2^-∆∆Ct^ method. All experiments were performed independently at least three times. The primers used were the following: *TNF-α*, (FW) 3′–CCCCAGTCTGTATCCTTCTA–5′ and (RV) 3′–CACTGTCCCAGCATCTTGT; *IL-1β*, (FW) 3′–AAGGGCTGCTTCCAAAC–5′ and (RV) 3′–TGTGCTGCTGCGAGATT–5′; *IL-6*, (FW) 3′–TACCACTCCCAACAGACC–5′ and (RV) 3′–TTTCCACGATTTCCCAGA–5′; *β-actin*, (FW) 3′–ATGTGGATCAGCAAGCAGGA–5′ and (RV) 3′–AAGGGTGTAAAACGCAGCTCA–5′; *CD206*, (FW) 3′–GCCGTCTGTGCATTTCCATTCAAG–5′ and (RV) 3′–TTTGTCGTAGTCAGTGGTGGTTCC–5′; *ARG1*, (FW) 3′–GTGAGAGACCACGGGGACCTG–5′ and (RV) 3′–CCACACCAGCCAGCTCTTCATTG–5′; *iNOS*, (FW) 3′–ACAGGAACCTACCAGCTCACTCTG–5′ and (RV) 3′–ACCACTGGATCCTGCCGATGC–5′; *IL-10*, (FW) 3′–CTGCTATGCTGCCTGCTCTTACTG–5′ and (RV) 3′–TGGGAAGTGGGTGCAGTTATTGTC–5′; *TGF-β*, (FW) 3′–ACTTGCACCACGTTGGACTTCG–5′ and (RV) 3′–TGGGTCATCACCGATGGCTCAG–5′; *MAP2*, (FW) AAGGCACCTCACTGGACCTCAG–5′ and (RV) ACCCTCTTCATCCTCCCTGTATGG–5′; *NeuN*, (FW) 3′–AGACAGACGAGGCGGCACAG–5′ and (RV) 3′–AGGGGATGTTGGAGACGTGTAGC–5′.

For western blot, equal amounts of protein were extracted using RIPA buffer (Sigma-Aldrich) and separated in SDS polyacrylamide gel. After electrophoresis, the proteins were transferred onto polyvinylidene difluoride membranes, which were then blocked with milk for 1 h. Afterwards, the proteins were labeled with the following primary antibodies overnight at 4° C: anti-MAP2, anti-CD206, anti-CD11b, anti-IBA-1, anti-β-actin (ProteinTech, Chicago, IL, USA) and anti-NeuN (Cell Signaling Technology, Danvers, MA, USA). After washing, the membranes were incubated for 1 h with a fluorescently labeled secondary antibody (1:5,000; Thermo Fisher Scientific, Waltham, MA, USA). β-actin was used as internal reference. The labeled proteins were observed using Odyssey (LI-COR Biosciences, Lincoln, NE, USA). ImageJ software (National Institutes of Health, Bethesda, MD, USA) was used for quantitative analysis of the protein bands.

### Statistical analysis

Statistical analyses were conducted using SPSS software v.16 (SPSS Inc., Chicago, IL, USA) and analysis of variance was used. All results were considered significant at *p* ≤ 0.05 and expressed as mean ± standard deviation (SD, n = 6). Image analysis was performed using GraphPad Prism v.6 (GraphPad Software, San Diego, CA, USA).

## RESULTS

### Characterization and toxicity of MIONs@PDA

**Figure 1:**
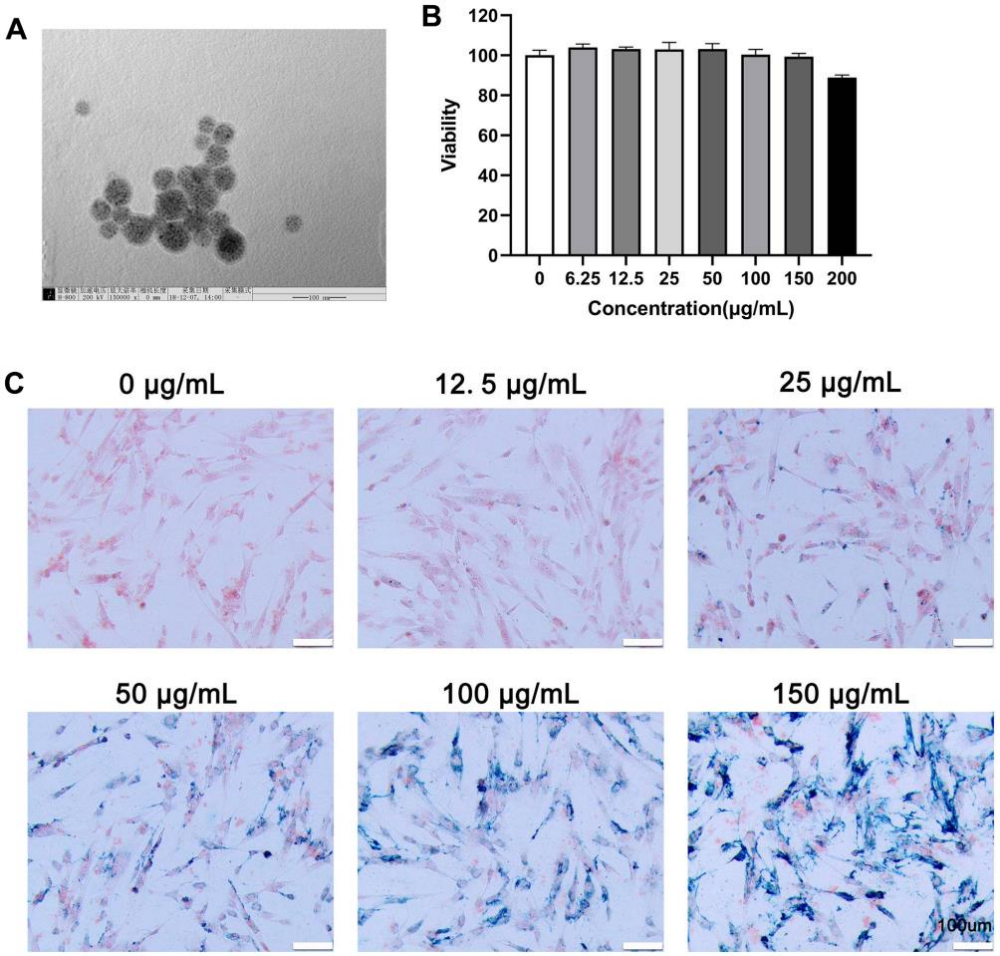
**Characterization, viability, and internalization potential of polydopamine-capped Fe_3_O_4_ nanoparticles (MIONs@PDA).** (**A**) Transmission electron microscopy imaging of MIONs@PDA. Scale bar = 50 nm. (**B**) Proliferation of human umbilical cord mesenchymal stem cells (HUMSCs) labeled with MIONs@PDA at concentrations of 0, 6.25, 12.5, 25, 50, 100, 150, and 200 μg/mL by Cell Counting Kit-8 assay. (**C**) Morphology of HUMSCs labeled with the MIONs@PDA at concentrations of 0, 25, 50, 75, 100, and 150 μg/mL. Scale bars = 100 μm.

Nanoparticles larger than 100 nm can scarcely penetrate cells by cellular phagocytosis. TEM images showed that the average diameter of MIONs was about 45–50 nm, which slightly increased to 50–60 nm after evenly encapsulated within the PDA, with MIONs@PDA being appropriately sized nanoparticles for labeling cells (Figure 1A). Viability experiments indicated that different concentrations of MIONs@PDA had a small negative effect on HUMSCs (Figure 1B). To investigate the internalization potential of MIONs@PDA by HUMSCs, Prussian blue staining was performed (Figure 1C), revealing that 50 μg/mL MIONs@PDA efficiently induced blue-stained deposits and cell labeling.

### Characteristics of HUMSCs labeled with MIONs@PDA

**Figure 2:**
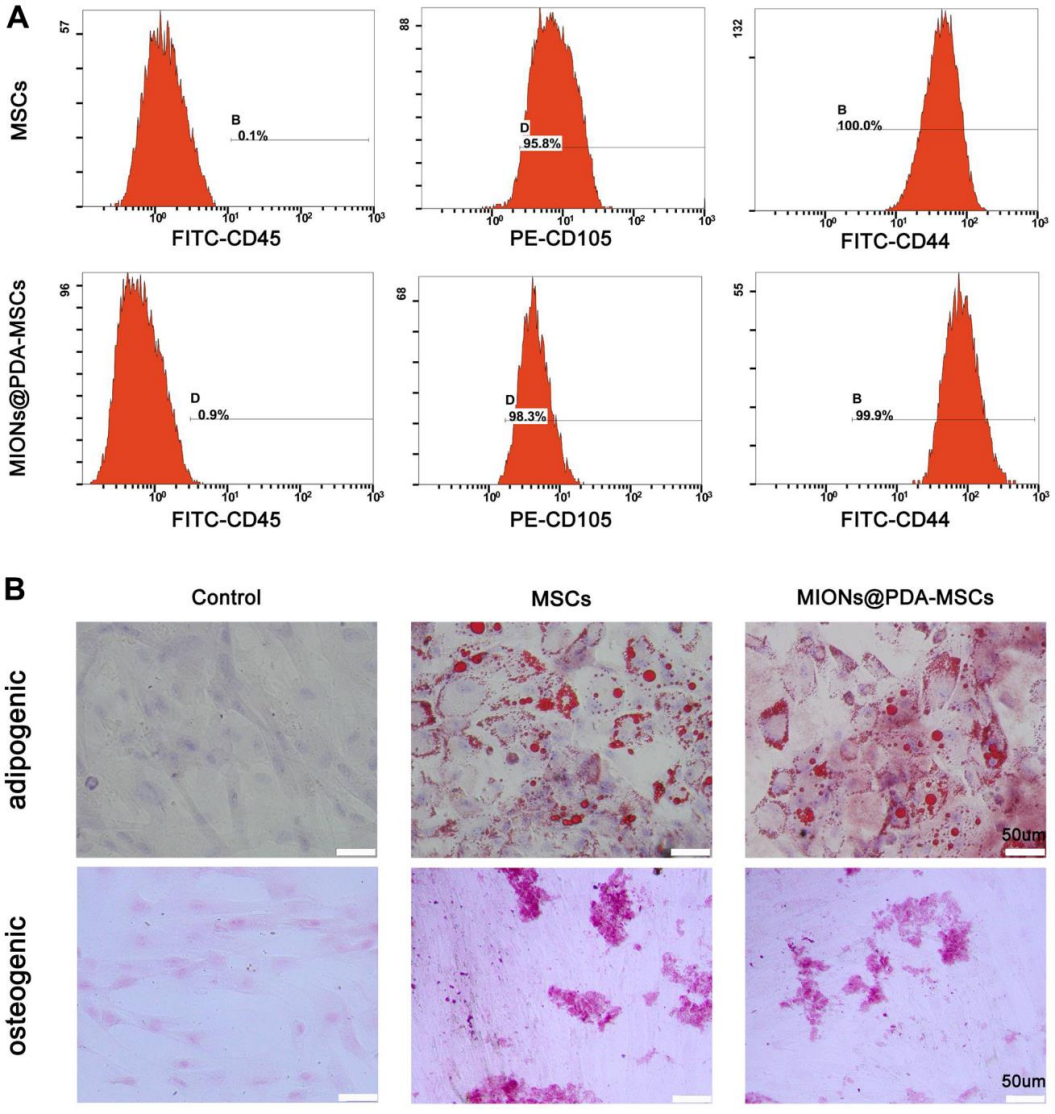
**Characterization of human umbilical cord mesenchymal stem cells (HUMSCs) labeled or not with polydopamine-capped Fe_3_O_4_ nanoparticles (MIONs@PDA).** (**A**) Similar to normal HUMSCs, MIONs@PDA-MSCs highly expressed the typical surface markers CD44 and CD90, but not the hematopoietic cell marker CD45. (**B**) Osteocyte and adipocyte differentiation of MIONs@PDA-MSCs vs. control HUMSCs. All cells exhibited adipogenic and osteogenic differentiation potential similar to that of control HUMSCs. Scale bars = 50 μm.

To determine whether the MIONs@PDA-MSCs did not lose differentiation potential, control HUMSCs and MIONs@PDA-MSCs were subjected to a differentiation assay. Von Kossa, Oil red O, and Alcian blue staining confirmed that both MIONs@PDA-labeled and unlabeled HUMSCs maintained their differentiation potential into osteocyte and adipocyte, respectively. Flow cytometry analysis showed that cultured HUMSCs and MIONs@PDA-MSCs highly expressed the cell surface markers of typical MSCs (cluster of differentiation CD105: 95.8 ± 1.7%, CD44: 100 ± 0.1%) but not hematopoietic cell markers (CD45: 0.1 ± 0.1%) (Figure 2A). Differences noted between the control and labeled cells were without statistical significance. These results indicated that the MIONs do not affect the characteristics of the HUMSCs. Therefore, these cells were used in the subsequent experiments (Figure 2B).

### Ability of HUMSCs to target the lesion region and reduce the volume of the infarct zone

**Figure 3:**
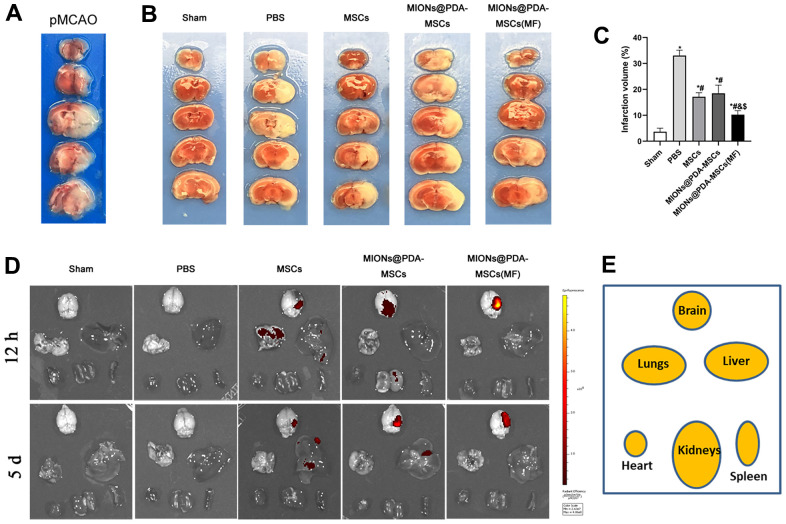
**Effects of HUMSCs on infarct volume and behavioral improvement.** (**A**) Representative brain slices with infarction volume shown by TTC staining. (**B**, **C**) HUMSCs treatment significantly reduced infarct volume. (**D**, **E**) Bio-distribution of the MSCs following their intravenous injection into the pMCAO-induced mice with or without the MF, evaluated by the IVIS imaging of major organs. Data are presented as the means ± standard deviation. HUMSC, human umbilical cord mesenchymal stem cell; MIONs@PDA-MSCs, HUMSCs labeled with polydopamine-capped Fe_3_O_4_ nanoparticles; MIONs@PDA-MSC(MF), MIONs@PDA-MSCs with external magnetic field; PBS, middle cerebral artery occlusion with phosphate-buffered saline administration; Sham, sham operation; TTC, 2,3,5-triphenyl-2H-tetrazoliuM chloride. ^*^p < 0.05 vs. Sham group, ^#^*p* <0 .05 vs. PBS group, ^&^*p* <0 .05 vs. HUMSCs group, ^$^*p* <0 .05 vs. MIONs@PDA-MSCs group.

To evaluate the therapeutic efficacy of transplanted HUMSCs at 5 d post-transplantation, a pMCAO *in vivo* model was achieved through surgery and infarct areas were confirmed based on TTC-stained brain sections. In this well-established animal model, the cortex mas the mainly affected region (Figure 3A). The percentage of infarct zone was significantly lower in all HUMSCs-transplanted mice than in the PBS group, and was significantly lower in mice treated with MIONs@PDA-MSCs(MF) group than in the other two groups. There was no significant difference between the HUMSCs and MIONs@PDA-MSCs treated groups (Figure 3B, 3C). For fluorescence imaging *in vivo*, MSCs were labeled with cm-dil before injection. Fluorescence imaging at 12 h after injection showed that in the absence of MF (MF-), a small amount of MSCs accumulated in the brain, whereas in the presence of MF, a large number of cells targeted the brain tissue. After 5 days, the fluorescence content in brain tissue of the magnetic target group was significantly higher than that of the non-MF group (Figure 3D, 3E).

### Histopathological changes induced by HUMSCs

**Figure 4:**
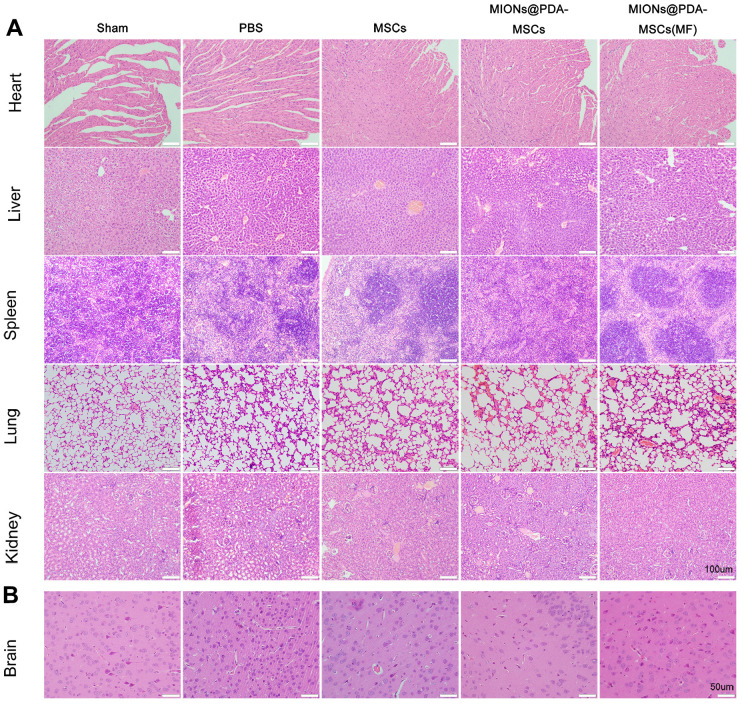
**Histopathological changes observed in different organs of mice in each group.** (**A**) The organs of each group were stained with hematoxylin and eosin. scale bars = 100 μm. (**B**) Pathological changes in cortical tissue. scale bars = 50 μm.

To evaluate the potential toxicity of the nanoparticles, hematoxylin and eosin staining showed no noticeable morphological changes in the heart, liver, spleen, lungs, and kidney in both treatment groups compared with the control group after 5 days of HUMSCs therapy, further indicating the low toxicity of the MIONs *in vivo*. As shown in Figure 4, cortical neurons and glial cells in brain tissues of the sham group were normally arranged and had normal structure. The cortical cells in the PBS group were disorganized, showing large areas of necrotic neurons, and glial cells proliferated significantly around the necrotic foci, with slight proliferation of small blood vessels. The neurons showed different degrees of ischemic changes, with the cytoplasm of the swollen neurons being tinged and having damaged membranes, whereas the shrunken neurons were deeply stained, with small and triangular cell bodies. The gap around the nerve cells and glial cells was widened, and the neurons were fixed and contracted. In comparison to the PBS group, neuronal and glial cell necrosis, cell membrane and cell structure destruction were significantly reduced after HUMSCs transplantation, and new small blood vessels were observed. In particular, most HUMSCs were magnetically targeted to the infarction area, where more residual neurons were and scattered glial cell proliferation was active.

### Stem cell differentiation potential

**Figure 5:**
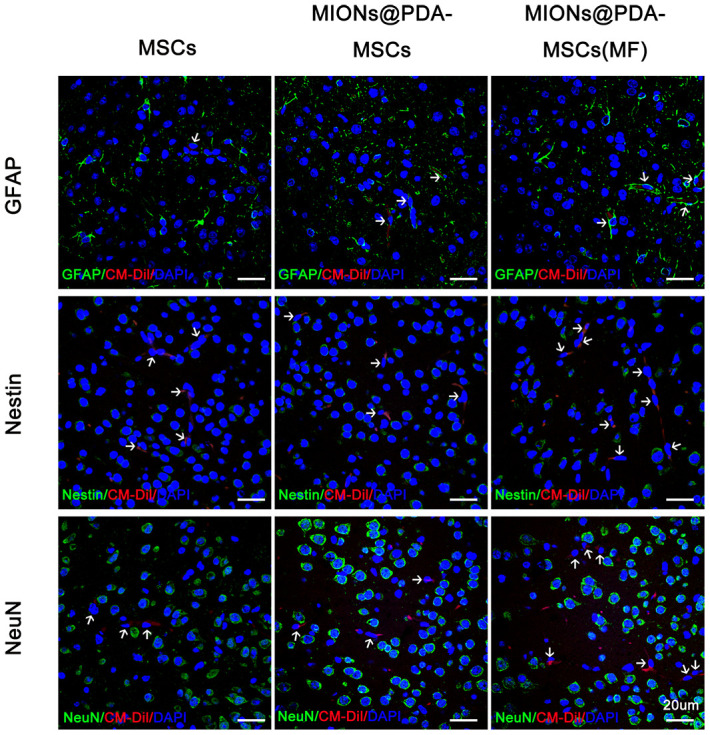
**Human umbilical cord mesenchymal cells (HUMSCS) differentiate into neural cells.** Immunofluorescence analysis showed no overlap between CM-Dil+HUMSCs and neurons (NeuN^+^), neurogliocyte (GFAP^+^), or neural stem cells (Nestin^+^). scale bars = 20 μm.

To verify whether HUMSCs could differentiate into neural cells, tissue slices are stained with different cells type markers: NeuN for neurons, GFAP for neurogliocyte and Nestin for neural stem cells. The results showed no overlap between CM-Dil+HUMSCs and NeuN^+^, GFAP^+^, or Nestin^+^ cells (Figure 5).

### Anti-inflammatory and neuroprotective effects of HUMSCs are promoted by the transition of microglia from M1 to M2 phenotype

**Figure 6:**
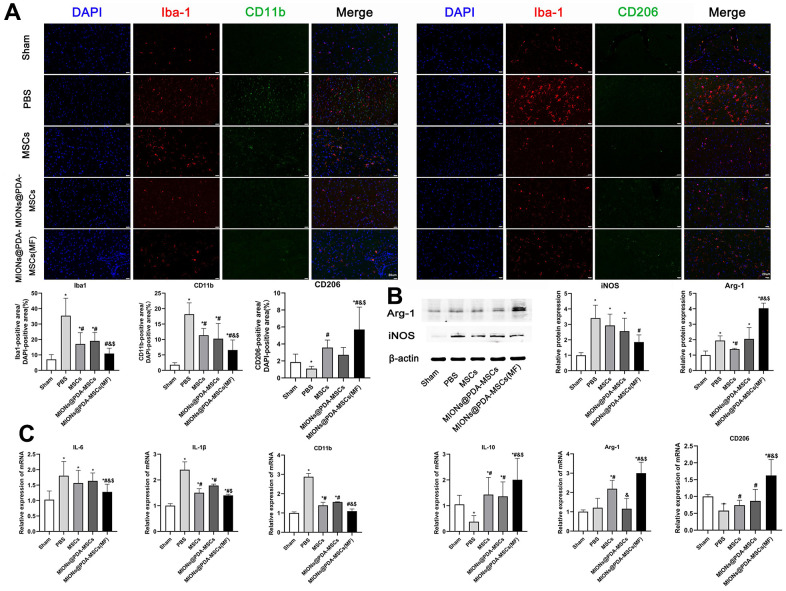
***In vivo* anti-inflammatory effects of the HUMSCs and MIONs@PDA-MSCs.** (**A**) Immunofluorescence analysis and quantification for M1 (CD11b) and M2 (CD206) macrophage markers in the cortex tissues of mice. scale bars=20 μm. (**B**) Western blot analysis and quantification of M1 (iNOS) and M2 (Arg-1) macrophage markers in the cortex tissues of mice. (**C**) Relative expressions of M1 (*IL-6, IL-1β* and *CD11b*) and M2 (*ARG-1, IL-10* and *CD206*) genes in the brain after treatment. *p < 0.05 vs. Sham group, ^#^*p* <0 .05 vs. PBS group, ^&^*p* <0 .05 vs. MSCs group, ^$^*p* <0 .05 vs. MIONs@PDA-MSCs group.

Next, it was investigated whether HUMSCs could exert enhanced anti-inflammatory effects *in vivo*. Immunofluorescence staining showed that Iba1^+^ microglia was increased in the cortex (infarct region) of mice treated with PBS. In comparison, HUMSCs inhibited pMCAO-induced microglia activation and MIONs@PDA-MSCs(MF) also revealed a clear inhibition (Figure 6A). qRT-PCR, immunofluorescence, and western blotting analysis of the brain following pMCAO were performed to study the effect of HUMSCs on pro-inflammatory and cytotoxic (M1), and anti-inflammatory and regenerative (M2) states (Figure 6). The mRNA expression of M1 macrophage markers (inducible nitric oxide synthase (iNOS), interleukin (IL)-1β, and tumor necrosis factor (TNF-α)), as well as M2 macrophage markers (arginase 1 (Arg-1), cluster of differentiation 206 (CD206), and IL-10) were evaluated (Figure 6C). The PBS group exhibited highly upregulated expression of the M1 markers, whereas the HUMSCs treatment significantly downregulated these genes, especially in the MIONs@PDA-MSCs(MF) group. In turn the M2 markers levels were considerably increased upon HUMSCs treatment. Western blotting and immunofluorescence analysis of M1 macrophage markers confirmed that the levels of CD11b and iNOS were increased after pMCAO, but reduced after HUMSCs transplantation, especially in the MIONs@PDA-MSCs(MF) group. The levels of M2 macrophage markers CD206 and Arg-1 were found to be decreased after pMCAO, but increased after HUMSCs transplantation, especially in the MIONs@PDA-MSCs(MF) group. These results suggest that HUMSCs can affect microglial phenotype transition to promote neurological functional recovery after pMCAO.

**Figure 7:**
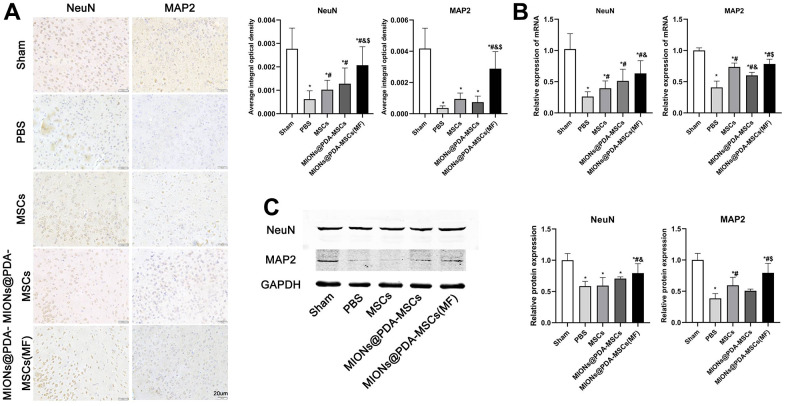
**HUMSCs exert neuroprotective effects *in vivo*.** (**A**) Immunofluorescence analysis, (**B**) relative mRNA expression, and (**C**) western blot analysis of neuronal markers (MAP2 and NeuN) in the cortex tissues of mice. ^*^p < 0.05 vs. Sham group, ^#^*p* <0 .05 vs. PBS group, ^&^*p* <0 .05 vs. MSCs group, ^$^*p* <0 .05 vs. MIONs@PDA-MSCs group. scale bars = 20 μm.

The effects of HUMSCs on cortical neurons survival were then investigated. The results showed that after pMCAO treatment, neuronal activity was significantly decreased and HUMSCs transplantation significantly improved neuronal survival, especially when more cells were targeted at the infarct site (Figure 7).

## DISCUSSION

MSCs were shown to protect several organs from damage and have been proposed as a promising strategy for patients with cerebral infarction who do not respond to other therapeutic strategies. Transplanted MSCs can migrate to the injury site and mediate tissue regeneration, primarily by the delivery of trophic and paracrine factors. As a result, *in vivo* persistence and secretory functions of transplanted MSCs are critical to the therapeutic outcome. Nevertheless, only few MSCs can effectively migrate to and engraft into the brain, with <0.001% of total administrated cells being able to survive and migrate to the infarcted cortex. Moreover, MSCs administrated to rodent models showed a higher mortality rate due to their tendency to adhere and aggregate, leading to capillary blockage [24]. Therefore, the main goal of this study was to improve the ability of stem cells to target and repair the site of cerebral infarction in mice.

To obtain MSCs with a good targeting capability, magnetic nanoparticles were prepared using PDA-capped Fe_3_O_4_. Since the 1970s, MIONs have been widely studied in the field of biomedicine, and their magnetic targeting properties have been developed for many biophysical and medical applications [25]. For example, MIONs bind to proteins, nucleotides, viral vectors, and immune and stem cells, and remotely control the distribution of drug molecules and cells in the body through MFs, to accumulate in target tissues [26, 27]. Indeed, MIONs, such as ferumoxytol, have been approved by the Food and Drug Administration for the treatment of iron-deficiency anemia. The high aggregation of Fe_3_O_4_ nanoparticles often impedes their uptake by cells [28]. Therefore, the present study used PDA encapsulation to reduce Fe_3_O_4_ aggregation and increase the nanoparticle uptake by MSCs. The results indicated that the PDA shell reduces the toxicity of the nanoparticles and greatly improved its superparamagnetism (the physiological stability and biocompatibility of Fe_3_O_4_ with the nucleus) [29]. Moreover, the collected data showed that 50 g/mL MIONs had minimal toxicity and good labeling effect on HUMSCs, without changing the characteristics and differentiation potential of the cells. HUMSCs internalized MIONs and mediated their magnetic navigation to the target infarction lesion in the brain with the assistance of an external MF. In this study, after more cells were targeted to the lesion site, the cerebral infarction volume was significantly reduced, and the pathological tissue was significantly repaired. In addition, the activity of glial cells was inhibited after MIONs@PDA-MSCs(MF) transplantation, and the survival activity of neurons was significantly improved in the magnetically targeted brain tissue. Therefore, MIONs@PDA-MSCs(MF) has great potential to repair cerebral infarction. However, MIONs@PDA-MSCs(MF) transplanted into mice were unable to differentiate into nerve cells; thus, HUMSCs may function through other mechanisms.

Microglia, as the resident immune cells of the central nervous system, can dynamically exist in surveillance (M0), pro-inflammatory (M1), and anti-inflammatory (M2) states [30–32]. Microglia are the first cells that respond to ischemic insult [33]. During the experimental infarction, the M1 phenotype largely increased in the peri-infarct regions and secreted pro-inflammatory mediators including TNF-α, IL-1β, and IL-6, and interferon (IFN)-γ that promote the secretion of reactive oxygen/nitrogen species and proteolytic enzymes, such as matrix metalloproteinase-9, which will in turn exacerbate inflammation and neuronal injury [34]. The phenotypic transition of M1 toward the M2 subtype is a promising therapeutic strategy for cerebral infarction [35]. It is recognized that the balance between M1 and M2 phenotypes determines the detrimental or beneficial effects of neuroinflammation on cerebral infarction. In the present, *in vivo* experiments showed that HUMSCs clearly promote the transformation of microglia from the M1 into the M2 phenotype. Moreover, CD11b^+^ M1 cells with amoeboid morphology were reduced, whereas CD206^+^ M2 microglia were promoted to exert anti-inflammatory function upon treatment with MION@PDA-MSC(MF). In particular, M2 microglia was found to protect the nervous tissues by the enhanced production of Arg-1, CD206, TGF-β, and IL-10.

In summary, this study revealed that MSCs targeted at the site of cerebral infarction can reduce the volume of cerebral infarction and promote microglial shift from the M1 to the M2 phenotype for neuroprotection and pro-neuroinflammation, thereby representing a potential novel approach for cerebral infarction therapy.
